# Association of Anterior Ventricular Aneurysm and D-Dimer with Intracardiac Thrombus in Patients with Acute Myocardial Infarction: A Retrospective Analysis

**DOI:** 10.3390/jcm15208173

**Published:** 2026-10-21

**Authors:** Bohan Li, Xiaorui Huang, Zizhu Lian, Shikang Liu, Tianyi Zhang, Tianjiao Lei, Guoliang Li, Yue Wu, Chaofeng Sun

**Affiliations:** 1 Department of Cardiovascular Medicine, The First Affiliated Hospital of Xi’an Jiaotong University, Xi’an 710061, China; xdtslbh@xjtu.edu.cn (B.L.); huangxr2025@163.com (X.H.); lsk@xjtufh.edu.cn (S.L.); liguoliang_med@163.com (G.L.); 2 Department of Cardiovascular Surgery, The First Affiliated Hospital of Xi’an Jiaotong University, Xi’an 710061, China; lisalzz2651092799@163.com (Z.L.); ltj585@xjtufh.edu.cn (T.L.); 3 Biobank, The First Affiliated Hospital of Xi’an Jiaotong University, Xi’an 710061, China; zhantianyi850214@xjtufh.edu.cn

**Keywords:** acute myocardial infarction, ventricular aneurysm, intracardiac thrombus, D-dimer, echocardiography

## Abstract

**Background:** Intracardiac thrombus is a clinically important complication of acute myocardial infarction (AMI), particularly in patients with ventricular aneurysm. We examined the contemporaneous associations of anterior ventricular involvement and D-dimer with echocardiographically documented intracardiac thrombus with incomplete chamber-specific localization among hospitalized AMI patients with ventricular aneurysm. **Methods:** This retrospective analysis included 1684 patients with documented ventricular aneurysm from a final cohort of 17,950 hospitalized AMI patients treated between January 2018 and October 2025. The first D-dimer measurement obtained at our hospital after AMI confirmation was used for D-dimer analyses. Ventricular aneurysm and intracardiac thrombus status were determined from the available echocardiographic documentation during the index hospitalization. Aneurysm locations were coded as linked patient-level, non-mutually exclusive indicators. Logistic regression evaluated anterior involvement; missing model covariates were multiply imputed and regression estimates were pooled using Rubin’s rules. Collinearity diagnostics and sensitivity analyses restricted to patients with apical aneurysm, including a complete-case analysis, were performed. Model-performance measures were interpreted as exploratory apparent performance in the development dataset. **Results:** Intracardiac thrombus was documented in 211 of 1684 patients (12.5%). Anterior involvement was more frequent among patients with thrombus than among those without thrombus (57.3% vs. 45.0%; *p* < 0.001) and remained associated with thrombus in the primary anatomical and clinical model (OR, 1.63; 95% CI, 1.20–2.21; *p* = 0.002). D-dimer was available in 1633 patients. On the log2-transformed scale, each doubling of D-dimer was associated with higher odds of echocardiographically documented intracardiac thrombus (adjusted OR, 1.24; 95% CI, 1.14–1.34; *p* < 0.001). As a complementary descriptive analysis, the observed thrombus proportion increased from 3.63% in Q1 to 22.30% in Q4; compared with Q1, the adjusted ORs were 3.71, 5.22, and 9.50 in Q2, Q3, and Q4, respectively. The combined model had a mean apparent AUC of 0.716, compared with 0.713 for the model containing D-dimer without anterior involvement; the mean absolute increment was approximately 0.003. **Conclusions:** Among hospitalized patients with AMI and ventricular aneurysm, higher D-dimer levels and anterior ventricular involvement were associated with echocardiographically documented intracardiac thrombus. Chamber-specific localization was incomplete, and these contemporaneous associations do not establish causality or prospective prediction. Anterior involvement provided minimal incremental discrimination beyond D-dimer.

## 1. Introduction

Intracardiac thrombus is a potentially serious complication of acute myocardial infarction (AMI) and remains clinically important in the contemporary reperfusion era [1,2,3,4]. Once formed, intracardiac thrombus may increase the risk of systemic embolism, stroke, and adverse cardiovascular outcomes [5,6]. Although advances in early reperfusion therapy and antithrombotic treatment have reduced the incidence of thrombotic complications after AMI, intracardiac thrombus remains clinically relevant, particularly in patients with extensive myocardial injury, impaired ventricular contractility, and ventricular aneurysm. Transthoracic echocardiography is widely used for initial assessment, but it remains uncertain which patients require intensified or repeat imaging when the initial examination is negative or equivocal [7,8,9]. This uncertainty supports investigation of the contemporaneous associations of anatomical findings and biomarker levels with echocardiographically documented intracardiac thrombus.

Ventricular aneurysm represents a structural consequence of myocardial infarction and is characterized by localized ventricular wall thinning, dyskinesia, or akinesia. The formation of a ventricular aneurysm may promote blood stasis and local flow disturbance, thereby creating a substrate for thrombus formation. However, ventricular aneurysm is not a homogeneous condition. Aneurysm location may influence intracardiac hemodynamics and thrombotic risk. In particular, anterior ventricular aneurysm may reflect larger infarct burden, greater involvement of the left ventricular cavity, and more pronounced regional wall motion abnormality, which may collectively increase the likelihood of thrombus formation [7,10,11,12].

In addition to structural abnormalities, systemic coagulation activation may contribute to intracardiac thrombus. Existing risk-assessment studies have primarily emphasized infarct territory, left ventricular dysfunction, and imaging features detected by echocardiography or cardiac magnetic resonance. D-dimer, a fibrin degradation product, reflects fibrin formation and degradation [13,14] and is routinely available in acute-care practice. Elevated D-dimer has been associated with thrombotic burden and adverse outcomes in cardiovascular disease and acute coronary syndromes [15]. We therefore evaluated D-dimer as a pragmatic biomarker that could complement anatomical assessment; whether it provides incremental value beyond clinical features and aneurysm location in AMI patients with ventricular aneurysm remains insufficiently defined.

Therefore, using retrospectively collected clinical records from hospitalized patients with AMI, we aimed to examine the contemporaneous associations of anterior ventricular involvement and D-dimer with echocardiographically documented intracardiac thrombus among patients with ventricular aneurysm. We also performed exploratory comparisons of apparent model performance; these analyses were not intended to guide clinical decision-making or determine the need for additional imaging.

## 2. Materials and Methods

### 2.1. Study Design and Population

This single-center retrospective analysis used clinical records from hospitalized patients with AMI treated at the First Affiliated Hospital of Xi’an Jiaotong University between January 2018 and October 2025. AMI diagnosis and clinical classification followed contemporary universal-definition and acute coronary syndrome guideline concepts [16,17]. The final AMI cohort comprised 17,950 unique patients. A unique patient identifier was used to identify repeated hospitalizations; repeated admissions were not counted as separate participants, and the first eligible AMI hospitalization during the study period was used as the index hospitalization.

All 17,950 patients had echocardiographic documentation sufficient for classification of recorded ventricular aneurysm and intracardiac-thrombus status in the clinical database. No patients were excluded on the basis of echocardiographic image quality or completeness; classification was based on the echocardiographic documentation available in the clinical database. There were no age restrictions, all AMI types were eligible, and no additional clinical or demographic exclusion criteria were applied. Previous myocardial infarction and potentially pre-existing ventricular aneurysm were not exclusion criteria because aneurysm chronicity could not be determined reliably from the available records.

Ventricular aneurysm status and location were derived from available echocardiographic or clinical documentation rather than centralized image re-adjudication or systematic CMR/CT confirmation. Ventricular aneurysm was defined as a discrete, persistent regional outpouching or contour deformity present in both end-diastole and end-systole, with myocardial thinning or remodeling and akinetic or dyskinetic systolic motion. Among the final cohort, 1684 patients had documented ventricular aneurysm and constituted the primary analytic cohort; 211 had documented intracardiac thrombus and 1473 did not.

### 2.2. Data Collection and Variables

Demographic characteristics, vital signs, medical history, laboratory measurements, cardiac biomarkers, renal function markers, coagulation parameters, inflammatory biomarkers, and echocardiographic findings were extracted from the clinical database. Demographic and clinical variables included age, sex, systolic blood pressure, diastolic blood pressure, pulse rate, history of hypertension, history of diabetes mellitus, history of atrial fibrillation, and prior stroke. Laboratory variables included N-terminal pro-B-type natriuretic peptide (NT-proBNP), high-sensitivity C-reactive protein (hs-CRP), fibrinogen, international normalized ratio (INR), prothrombin activity, albumin, white blood cell count, neutrophil count, lymphocyte count, creatinine, estimated glomerular filtration rate (eGFR), glucose, creatine kinase-MB (CK-MB), cardiac troponin T (cTnT), and D-dimer. For D-dimer and CK-MB, the first result obtained at our hospital after AMI confirmation was used. D-dimer was recorded in mg/L. The analytic dataset did not contain verified assay-platform details, reagent information, local reference intervals, FEU/DDU reporting conventions, or records of laboratory platform changes during 2018–2025; these details were therefore not inferred. D-dimer assays may use different analytical platforms and may report fibrin-equivalent units or D-dimer units, which are not directly interchangeable. The observed quartile boundaries were recalculated from patient-level values and were interpreted as internal descriptive cut points rather than validated clinical thresholds. The observed D-dimer quartile boundaries were 0.59, 0.91, and 1.80 mg/L. Quartile-based and natural-spline analyses were restricted to patients with observed D-dimer measurements, and imputed D-dimer values were not used to define quartile groups or dose–response curves. Left ventricular systolic function grade, as recorded in the source echocardiographic report, was retained only as an ordinal adjustment covariate in the multivariable models and was not reported as a standalone descriptive or screening variable. Ventricular aneurysm location was classified from echocardiographic or clinical documentation as apical, anterior, inferior, septal, lateral, posterior, or right ventricular involvement, as applicable. Location variables were non-mutually exclusive because an individual patient could have aneurysmal involvement of more than one ventricular segment. Accordingly, location-specific analyses were interpreted as segment-involvement analyses rather than comparisons between mutually exclusive anatomical groups.

### 2.3. Outcome

The primary outcome was echocardiographically documented intracardiac thrombus during the index hospitalization. The first echocardiographic examination performed at our hospital after AMI confirmation was used for initial assessment of ventricular aneurysm status and chamber findings. Intracardiac thrombus was recorded when it was documented in the available echocardiographic record during the index hospitalization [18]. A thrombus documented only on a later repeat examination was classified as detected during the index hospitalization and was not backdated to the first echocardiographic examination; the analysis did not treat a negative initial examination as positive.

Cardiac magnetic resonance and cardiac computed tomography were not used systematically to confirm the outcome. The analytic dataset did not retain a complete examination-level inventory identifying conventional transthoracic echocardiography, contrast-enhanced echocardiography, or transesophageal echocardiography for every examination. Transesophageal echocardiography was therefore not separately analyzed, and we cannot determine whether any event was detected exclusively by TEE. A negative echocardiographic assessment was not considered an independent reference-standard negative result.

Because chamber-specific thrombus localization was not consistently recorded, the outcome was not restricted to exclusively confirmed left ventricular thrombus. Available clinical records suggested that many documented thrombi involved the left ventricle; however, the proportion and distribution of thrombi among the left ventricle, right ventricle, atria, and other cardiac chambers could not be verified. No systematic central image re-adjudication, pathology confirmation, or standardized follow-up confirmation was available to exclude all non-thrombotic cardiac masses that might mimic thrombus.

### 2.4. Statistical Analysis

Continuous variables were presented as median and interquartile range and compared using the Mann–Whitney U test. Categorical variables were presented as number and percentage and compared using the chi-square test or Fisher’s exact test, as appropriate. Logistic regression models were used to estimate odds ratios (ORs) and 95% confidence intervals (CIs) for the association between aneurysm location and intracardiac thrombus. Because location variables were non-mutually exclusive, models evaluating anterior ventricular aneurysm adjusted sequentially for other documented aneurysm locations and clinical or laboratory variables. D-dimer measurements were available in 1633 of 1684 patients with ventricular aneurysm. Missing values in model covariates were handled under a missing-at-random assumption using multiple imputation by chained equations. Twenty imputed datasets were generated, with 20 iterations performed for each dataset. Binary variables were imputed using logistic regression, and continuous variables were imputed using predictive mean matching. The outcome indicator and all prespecified model variables were included as predictors in the imputation models. Log2-transformed D-dimer, log2-transformed NT-proBNP, and log2-transformed hs-CRP [log2(hs-CRP + 1)] were generated before imputation and imputed on the transformed scale. A ridge parameter of 1 × 10^−4^ was used to improve numerical stability. Visual inspection of trace plots and density plots did not show obvious evidence of non-convergence or substantial incompatibility between observed and imputed distributions. Regression coefficients and their standard errors were pooled on the model-coefficient scale using Rubin’s rules, after which odds ratios and confidence intervals were obtained by exponentiation. This procedure retained all 1684 patients, including 211 thrombus events, in the multivariable analyses. Variable-specific missingness and imputation methods are summarized in Supplementary Table S13. For multivariable models that included D-dimer as a covariate, missing log2-transformed D-dimer values were imputed. In contrast, quartile-based and natural-spline analyses used observed D-dimer values only and therefore remained restricted to the 1633 patients with observed measurements.

For the primary D-dimer analysis, D-dimer was treated as a continuous exposure after log2 transformation, and odds ratios were interpreted per doubling of D-dimer. The quartile-based analysis was retained only as a complementary descriptive dose-gradient analysis. Patients with observed D-dimer measurements were divided into quartiles, and adjusted odds ratios were calculated using the lowest quartile as the reference group. The relationship between log2-transformed D-dimer and model-estimated thrombus probability was explored using natural-spline modeling. No formal test for nonlinearity was prespecified.

To evaluate the influence of extreme recorded D-dimer values, the continuous log2-transformed D-dimer model was additionally refitted among patients with observed D-dimer measurements under four exploratory scenarios: exclusion of the upper 1% of values, winsorization at the 99th percentile, exclusion of recorded values >100 mg/L, and exclusion of recorded values >1000 mg/L. The same outcome definition, covariate specification, and model estimation approach as in the continuous D-dimer analysis were used. Where applicable, missing values in other model covariates were handled using the same multiple-imputation procedure as in the primary analyses. These thresholds were used as exploratory stress-test definitions and were not interpreted as assay-specific clinical cutoffs. Models 1–3 were used as the primary anatomical and clinical association analyses. Model 4 additionally incorporated D-dimer and other laboratory markers and was considered an extended exploratory model. Because D-dimer was measured during the same hospitalization and its temporal relationship to thrombus detection was not standardized, Model 4 was not interpreted as a causal fully adjusted model or as establishing an anatomically independent effect.

As a calendar-time sensitivity analysis, the prespecified adjusted continuous log2-transformed D-dimer model was fitted in the full cohort with observed D-dimer measurements and repeated after excluding patients admitted in 2025. The same outcome definition, D-dimer specification, covariate set, missing-data procedure, and model-performance calculations were used in both analyses; the only difference was the exclusion of patients admitted in 2025. Admission year was determined from the hospital admission date. The resulting estimates were interpreted descriptively as a calendar-time sensitivity analysis and were not used to establish assay comparability across calendar years. This sensitivity model was distinct from the simpler Base + log D-dimer model used for the model-performance comparison.

For each imputed dataset, AUC, Brier score, AIC, and BIC were calculated separately for each prespecified model. These quantities were summarized as arithmetic means and standard deviations across the 20 imputed datasets. DeLong comparisons were performed separately within each imputed dataset to compare each expanded model with the base clinical model using the DeLong method [19]. Because DeLong *p* values were not formally pooled across imputations, they were summarized descriptively as ranges and were not interpreted as pooled inferential estimates. All model-performance measures were considered exploratory apparent-performance measures obtained in the development dataset. Model reporting and interpretation were guided by recommendations for observational and prediction-model studies [20,21], and overall model-performance assessment was informed by established prediction-model methodology [22,23]. A two-sided *p* value < 0.05 was considered statistically significant.

Aneurysm-location variables were available as linked patient-level indicators. The anterior-by-apical cross-tabulation was therefore calculated directly. Because anterior and apical involvement were non-mutually exclusive and frequently co-occurred, their association was assessed using the phi coefficient and variance inflation factors (VIFs). The phi coefficient was 0.142, and the maximum VIF was 2.28, indicating no severe multicollinearity by conventional thresholds. Additional sensitivity analyses were performed in the apical-aneurysm subgroup, in which the apical indicator was omitted because it was invariant, and in complete cases. The first D-dimer measurement and the first echocardiographic examination obtained at our hospital after AMI confirmation were selected for analysis. However, the timing of D-dimer sampling relative to the first examination that documented thrombus was not consistently available. Therefore, the analysis does not establish that D-dimer elevation preceded thrombus formation, and a sensitivity analysis restricted to D-dimer measurements obtained before thrombus detection could not be performed.

## 3. Results

**Figure 1:**
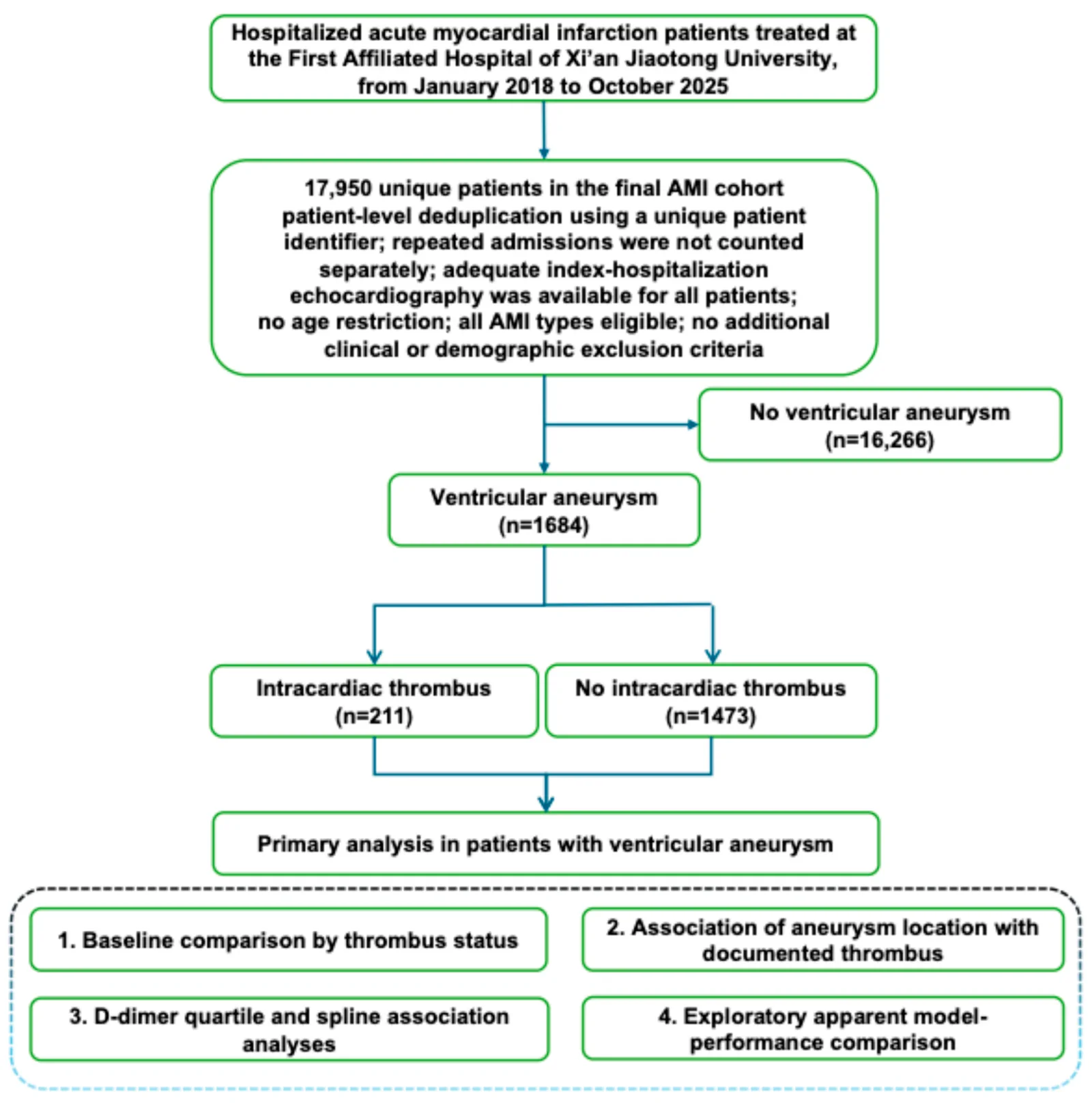
Flowchart of patient-level cohort derivation and analytic groups. The study included 17,950 unique patients hospitalized with AMI between January 2018 and October 2025. Repeated admissions were identified using a unique patient identifier and were not counted separately. All patients had echocardiographic documentation during the index hospitalization sufficient for classification of documented ventricular aneurysm and intracardiac-thrombus status in the clinical database. Of these patients, 16,266 had no documented ventricular aneurysm and 1684 had documented ventricular aneurysm; the latter included 211 patients with and 1473 without echocardiographically documented intracardiac thrombus.

A total of 17,950 patients hospitalized with AMI were included in the final cohort. Repeated admissions were identified using a unique patient identifier and were not counted as separate participants. All patients had echocardiographic documentation sufficient for classification of documented ventricular aneurysm and intracardiac-thrombus status in the clinical database. No additional age, AMI-type, clinical, or demographic exclusion criteria were applied. Among these patients, 16,266 had no documented ventricular aneurysm and 1684 had documented ventricular aneurysm. Within the ventricular aneurysm cohort, 211 patients had echocardiographically documented intracardiac thrombus and 1473 had no documented thrombus. The primary analysis was restricted to patients with documented ventricular aneurysm. The cohort derivation and analytic groups are summarized in Figure 1.

Baseline characteristics of patients with ventricular aneurysm stratified by intracardiac thrombus status are shown in Table 1. Patients with intracardiac thrombus were younger than those without thrombus [61.00 (52.00, 70.00) years vs. 64.00 (56.00, 72.00) years, *p* < 0.001] and were more frequently male [181 (85.8%) vs. 1,090 (74.0%), *p* < 0.001]. Pulse rate was higher in patients with thrombus [86.00 (76.00, 98.00) vs. 83.00 (73.00, 95.00) beats/min, *p* = 0.002]. NT-proBNP, hs-CRP, fibrinogen, INR, and creatinine were significantly higher in patients with thrombus, whereas prothrombin activity and glucose were lower. The absolute median difference in hs-CRP was small after rounding despite statistical significance.

Aneurysm locations were non-mutually exclusive, linked patient-level indicators. The exact anterior-by-apical cross-tabulation and collinearity diagnostics were calculated from patient-level data and are provided in Supplementary Tables S5 and S6. The phi coefficient was 0.142 and the maximum VIF was 2.28. Additional sensitivity analyses restricted to patients with apical aneurysm and complete cases are provided in Supplementary Tables S7 and S8. The comparison of each aneurysm location with patients without any ventricular aneurysm was retained only as a secondary descriptive analysis and is presented in Supplementary Table S1.

**Figure 2:**
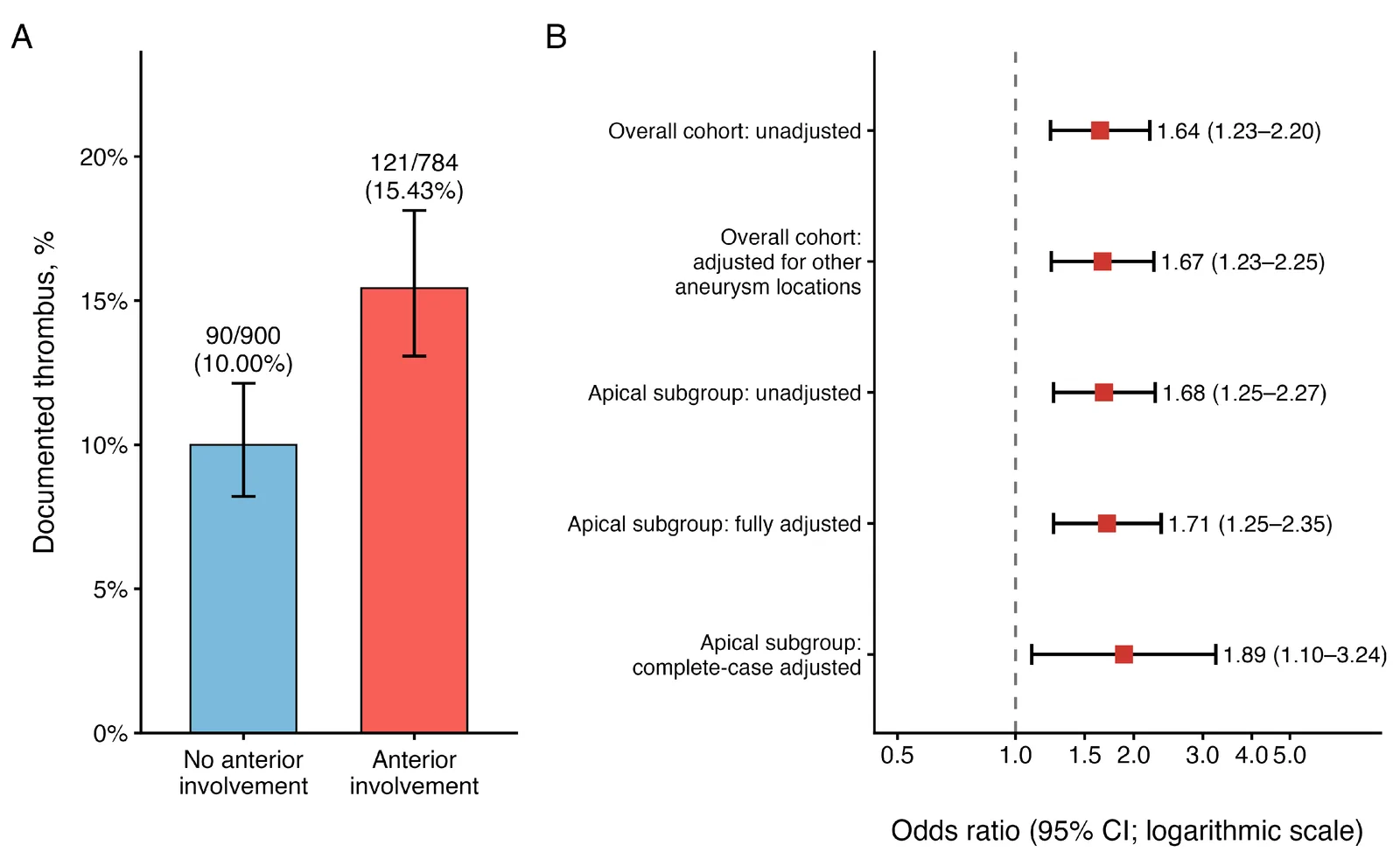
Association of anterior ventricular aneurysm involvement with echocardiographically documented intracardiac thrombus among patients with ventricular aneurysm. (**A**) Observed proportions of echocardiographically documented intracardiac thrombus among patients without and with anterior ventricular aneurysm involvement: 90/900 (10.00%) and 121/784 (15.43%), respectively. Error bars represent 95% confidence intervals for the observed proportions. (**B**) Odds ratios (ORs) and 95% confidence intervals (CIs) for the association between anterior ventricular aneurysm involvement and echocardiographically documented intracardiac thrombus in the overall ventricular aneurysm cohort and in the subgroup with apical aneurysm. Overall-cohort models included an unadjusted model and sequential multivariable models adjusted for other aneurysm locations, clinical variables, and laboratory variables. The apical-subgroup models evaluated the association of additional anterior involvement after omitting the invariant apical indicator. Because aneurysm-location indicators were non-mutually exclusive and anterior and apical involvement frequently co-occurred, these estimates represent conditional associations rather than anatomically isolated or causal effects. The vertical dashed line indicates OR = 1.

Within the ventricular aneurysm cohort, thrombus was documented in 121 of 784 patients with anterior ventricular involvement (15.43%) and in 90 of 900 patients without anterior involvement (10.00%). The observed proportions are shown in Figure 2A, and the corresponding unadjusted and adjusted ORs are shown in Figure 2B. The corresponding unadjusted OR was 1.64 (95% CI, 1.23–2.20; *p* < 0.001; Supplementary Table S4). After adjustment for other aneurysm locations, the OR was 1.67 (95% CI, 1.23–2.25; *p* < 0.001). After further adjustment for clinical variables and other aneurysm locations, the OR was 1.63 (95% CI, 1.20–2.21; *p* = 0.002). In the primary anatomical and clinical association model, anterior ventricular involvement remained associated with echocardiographically documented intracardiac thrombus (OR, 1.63; 95% CI, 1.20–2.21; *p* = 0.002). In the extended exploratory model that additionally included D-dimer and other laboratory markers, the association remained similar (OR, 1.65; 95% CI, 1.20–2.26; *p* = 0.002). Because anterior and apical involvement were non-mutually exclusive and frequently co-occurred, this estimate should be interpreted as a conditional association within the ventricular aneurysm cohort rather than as an anatomically isolated or causal effect. The corresponding patient-level anterior-by-apical cross-tabulation, collinearity diagnostics, apical-subgroup analysis, and complete-case sensitivity analysis are provided in Supplementary Tables S5–S8. Because anterior and apical involvement substantially overlapped, this comparison primarily contrasts patients with combined apical-plus-anterior involvement with those having apical involvement without anterior involvement. It should not be interpreted as evidence for an isolated anterior anatomical effect.

The first D-dimer result obtained at our hospital after AMI confirmation was available in 1633 patients, including 208 thrombus events. Multiple imputation of missing model covariates allowed the multivariable analyses to include all 1684 patients and 211 events. The observed-value quartile and natural-spline analyses remained restricted to the 1633 patients with measured D-dimer, and imputed D-dimer values were not used to define quartile groups.

**Figure 3:**
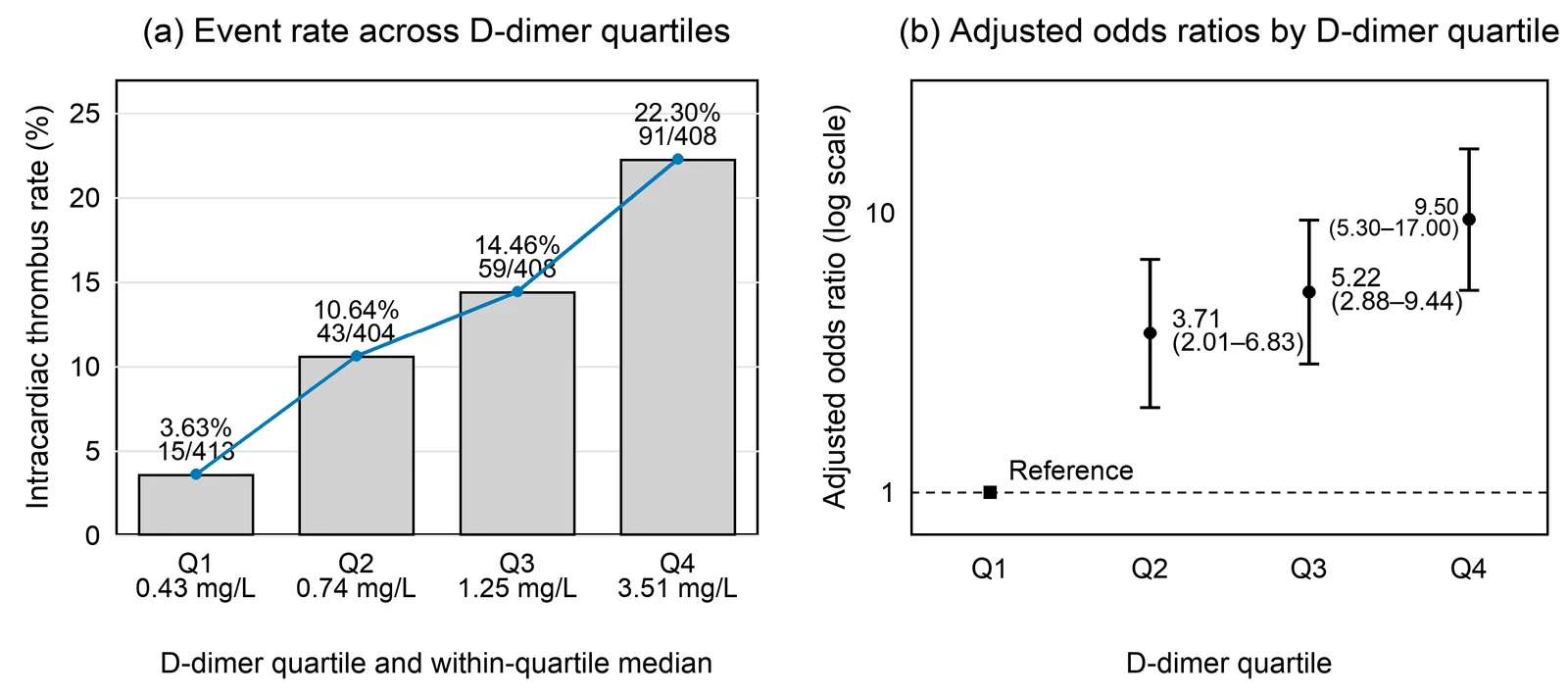
Association between quartiles of the first D-dimer measurement obtained at our hospital after AMI confirmation and echocardiographically documented intracardiac thrombus among patients with ventricular aneurysm. (**a**) Observed proportions of patients with echocardiographically documented intracardiac thrombus across D-dimer quartiles, with event counts, total counts, and corresponding 95% confidence intervals. The x-axis displays the quartile group and the within-quartile median D-dimer value; these medians are descriptive and are not the exact quartile cut points. (**b**) Adjusted odds ratios (ORs) and 95% confidence intervals (CIs) for echocardiographically documented intracardiac thrombus across D-dimer quartiles, with Q1 as the reference. Analyses included 1633 patients with observed D-dimer values; imputed values were not used to define quartiles. The quartile boundaries were 0.59, 0.91, and 1.80 mg/L and represent descriptive, cohort-specific cut points rather than validated clinical thresholds. Models were adjusted for prespecified clinical, echocardiographic, aneurysm-location, and laboratory covariates, with missing covariates handled by multiple imputation. Estimates are exploratory associations.

Before imputation, the observed proportion of patients with documented thrombus was 5.88% (3/51) among patients with missing D-dimer and 12.74% (208/1633) among those with available D-dimer (two-sided Fisher’s exact *p* = 0.196). This outcome-only comparison does not establish comparability of baseline characteristics between the groups. On the log2-transformed scale, each doubling of D-dimer was associated with higher odds of echocardiographically documented intracardiac thrombus (adjusted OR, 1.24; 95% CI, 1.14–1.34; *p* < 0.001; Supplementary Table S10). The quartile-based analysis was presented as a complementary descriptive dose-gradient analysis. Consistent with this continuous association, the observed proportion of patients with documented thrombus increased across D-dimer quartiles: 15 of 413 patients (3.63%) in Q1, 43 of 404 (10.64%) in Q2, 59 of 408 (14.46%) in Q3, and 91 of 408 (22.30%) in Q4 (Supplementary Table S2 and Figure 3a). Using Q1 as the reference, adjusted ORs increased stepwise: 3.71 (95% CI 2.01–6.83, *p* < 0.001) in Q2, 5.22 (95% CI 2.88–9.44, *p* < 0.001) in Q3, and 9.50 (95% CI 5.30–17.00, *p* < 0.001) in Q4 (Figure 3b). The natural-spline analysis was used as an exploratory visualization of the dose–response relationship and suggested possible curvature; no formal test for nonlinearity was prespecified or performed. The nonsignificant per-standard-deviation OR on the original scale was interpreted cautiously because the original-scale linear specification may not adequately represent the skewed distribution of D-dimer. It was not treated as evidence against the continuous log2-transformed association. The observed quartile boundaries were 0.59, 0.91, and 1.80 mg/L. The calendar-year distribution of D-dimer measurements is provided in Supplementary Table S9, and the exact quartile boundaries and continuous-analysis specifications are provided in Supplementary Table S10. These cut points were calculated from patient-level values within the present cohort and should not be interpreted as assay-independent clinical thresholds.

In sensitivity analyses restricted to patients with observed D-dimer measurements, the positive association between continuous log2-transformed D-dimer and intracardiac thrombus was preserved under all alternative extreme-value handling strategies. The OR per D-dimer doubling was 1.24 (95% CI, 1.14–1.34) using the original values, 1.48 (95% CI, 1.32–1.67) after excluding the upper 1%, 1.29 (95% CI, 1.18–1.41) after winsorization at the 99th percentile, 1.50 (95% CI, 1.32–1.70) after excluding values > 100 mg/L, and 1.30 (95% CI, 1.18–1.43) after excluding values > 1000 mg/L; all *p* values were <0.001 (Supplementary Table S11). These analyses did not change the direction or statistical significance of the association, although exclusion of extreme values yielded somewhat larger effect estimates.

In a calendar-time sensitivity analysis using the same prespecified adjusted model in both cohorts, the full cohort with observed D-dimer measurements included 1633 patients and 208 thrombus events. After excluding patients admitted in 2025, 1386 patients and 179 thrombus events remained. The OR per D-dimer doubling was 1.25 (95% CI, 1.15–1.35; *p* < 0.001) in the full cohort and 1.50 (95% CI, 1.31–1.72; *p* < 0.001) after exclusion of patients admitted in 2025. The corresponding mean apparent AUCs were 0.726 ± 0.003 and 0.749 ± 0.001, and the mean Brier scores were 0.1034 ± 0.0002 and 0.1021 ± 0.0002, respectively (Supplementary Table S12). The direction and statistical significance of the association were preserved, although the estimated effect size and apparent discrimination changed after exclusion of patients admitted in 2025. These measures were interpreted descriptively and not as evidence of assay comparability across calendar years.

Multivariable model performance is summarized in Table 2. Across the 20 imputed datasets, the base clinical model had a mean AUC of 0.635 ± 0.004. Adding anterior ventricular aneurysm increased the mean AUC to 0.649 ± 0.003, with a mean ΔAUC of 0.014 ± 0.001; the DeLong *p* values across imputations ranged from 0.125 to 0.252. Adding log-transformed D-dimer increased the mean AUC to 0.713 ± 0.003, with a mean ΔAUC of 0.078 ± 0.004; all corresponding DeLong *p* values were <0.001. The combined model had a mean AUC of 0.716 ± 0.003, with a mean ΔAUC of 0.081 ± 0.004 compared with the base model. Relative to the base plus D-dimer model, the combined model increased the mean AUC by approximately 0.003. The corresponding mean Brier scores were 0.1066, 0.1057, 0.1049, and 0.1040, respectively. These performance measures were summarized descriptively across the imputed datasets and were interpreted as exploratory apparent performance rather than as formally pooled or externally validated predictive performance.

## 4. Discussion

In this retrospective analysis of 1684 hospitalized AMI patients with documented ventricular aneurysm, we examined the contemporaneous associations of anterior ventricular involvement and D-dimer with echocardiographically documented intracardiac thrombus. The major findings were as follows. First, patients with thrombus had distinct clinical, coagulation, renal, and echocardiographic profiles. Second, anterior ventricular involvement was associated with intracardiac thrombus in the primary anatomical and clinical association model, and the estimate remained similar in the extended exploratory model that additionally included D-dimer and other laboratory markers. Third, D-dimer showed a strong graded association with thrombus, while the natural-spline analysis provided an exploratory visualization that suggested possible curvature; no formal test for nonlinearity was performed. Fourth, adding log-transformed D-dimer produced the largest apparent increase in discrimination among the models tested.

The present study highlights the potential importance of aneurysm location in thrombus formation after AMI, while also demonstrating the need for cautious interpretation of overlapping anatomical labels. Current AHA guidance emphasizes imaging-based assessment and suggests cardiac magnetic resonance when echocardiography raises concern for possible thrombus or clinical suspicion persists [4]. Recent reviews have also emphasized the expanding role of CMR in assessing ventricular morphology, function, tissue characterization, and intraventricular thrombus detection [24]. In a CMR meta-analysis, Bulluck et al. reported LV thrombus in 6.3% of all-comers STEMI patients, 12.2% of patients with anterior STEMI, and 19.2% of patients with anterior STEMI and LVEF < 50%; 96% of detected thrombi occurred after anterior STEMI [10]. That study also reported substantially lower sensitivity of routine transthoracic echocardiography than CMR. Direct comparison with our observed proportion is limited because our cohort was selected for documented ventricular aneurysm and used the broader outcome of intracardiac thrombus. These differences underscore how case selection, outcome definition, and imaging modality can influence observed prevalence. Our findings extend existing anatomical and imaging-based evidence by evaluating an accessible biomarker alongside aneurysm location, but they do not replace definitive imaging.

Several mechanisms may explain the association between additional anterior involvement and thrombus among patients with ventricular aneurysm. Anterior infarction often reflects extensive left anterior descending coronary artery territory injury and may extend toward the apex, where akinesia or dyskinesia, impaired emptying, and marked flow stasis favor thrombus formation. Apical remodeling or aneurysmal cavity distortion may further reduce washout [25]. These conditions, together with endocardial injury and systemic hypercoagulability, represent components of Virchow’s triad [26]. Given the substantial anterior–apical overlap, the analysis mainly compares combined apical-plus-anterior involvement with apical involvement alone. Therefore, the estimated association should be interpreted as conditional rather than as an isolated anatomical or causal effect.

D-dimer showed a graded association with echocardiographically documented intracardiac thrombus. On the log2-transformed scale, each doubling of D-dimer was associated with higher odds of documented thrombus (adjusted OR, 1.24; 95% CI, 1.14–1.34). The quartile-based analysis was included only as a complementary descriptive dose-gradient analysis; compared with Q1, the highest quartile was associated with approximately 9.5-fold higher adjusted odds of thrombus. The natural-spline analysis was used as an exploratory visualization of the dose–response relationship and suggested possible curvature; no formal test for nonlinearity was performed.

D-dimer reflects fibrin formation and degradation, and previous studies have linked elevated values to thrombotic burden and adverse outcomes in AMI and acute coronary syndrome populations [27,28]. However, D-dimer measurement and echocardiographic assessment were performed during the same hospitalization, and their precise temporal ordering was unavailable. Elevated D-dimer may therefore reflect fibrinolytic activity from thrombus that had already formed rather than a factor that preceded thrombus formation. Accordingly, D-dimer should be interpreted as a contemporaneous associated marker rather than as a prospective predictor, validated screening test, or stand-alone diagnostic test.

The development of intracardiac thrombus after AMI is likely influenced by both local anatomical factors and systemic biological activation. Ventricular aneurysm and anterior ventricular involvement reflect structural abnormality and flow disturbance, whereas D-dimer reflects coagulation and fibrin turnover. Adding log-transformed D-dimer to the base model yielded the largest apparent improvement in discrimination among the models tested; however, this was an exploratory development-sample comparison and does not establish clinical utility. Adding anterior ventricular aneurysm alone resulted in only a modest increase in AUC, with DeLong *p* values ranging from 0.125 to 0.252 across the 20 imputed datasets. The combined model had a mean AUC of 0.716 ± 0.003, compared with 0.713 ± 0.003 for the model containing D-dimer alone, corresponding to a mean absolute difference of approximately 0.003. The combined model also had a lower mean Brier score (0.1040 ± 0.0002 vs. 0.1049 ± 0.0002), AIC (1190.64 ± 2.70 vs. 1199.57 ± 2.58), and BIC (1234.08 ± 2.70 vs. 1237.57 ± 2.58) than the base plus D-dimer model. These differences were interpreted as exploratory apparent-performance findings rather than evidence of a clinically validated prediction model.

Clinically, ventricular aneurysm and D-dimer may provide contextual information when echocardiographic findings, image quality, ventricular function, and thrombotic and bleeding risks are interpreted together. However, these findings do not establish that D-dimer or anterior ventricular involvement should be used to determine additional imaging or management. D-dimer is nonspecific and should not by itself trigger a diagnosis or anticoagulation decision. Decisions regarding anticoagulation should be based on confirmed thrombus, embolic risk, bleeding risk, and the overall clinical context, consistent with the recognized challenges of left ventricular thrombus management and available evidence on antithrombotic therapy [29,30].

### Limitations

This study has several limitations. First, its retrospective single-center design is susceptible to residual confounding, selection bias, and limited generalizability. Second, ventricular aneurysm and intracardiac thrombus were identified from available clinical and echocardiographic documentation without central image re-adjudication. True aneurysm was not systematically distinguished from pseudoaneurysm or diverticulum, and aneurysm size, neck dimensions, quantitative regional wall-motion abnormality, quantitative LVEF, infarct size, coronary anatomy, reperfusion timing, and anticoagulation status were not consistently available.

Third, the primary outcome was echocardiographically documented intracardiac thrombus, and chamber-specific localization was incomplete. Available clinical context suggested that many documented thrombi involved the left ventricle, but the proportion and distribution of thrombi among the left ventricle, right ventricle, atria, and other intracardiac locations could not be verified. Cardiac magnetic resonance and cardiac computed tomography were not systematically performed for outcome confirmation, and examination-level information distinguishing conventional echocardiography, contrast-enhanced echocardiography, and transesophageal echocardiography was not consistently retained. Therefore, small or mural thrombi may have been missed, and the completeness of thrombus detection cannot be guaranteed. Non-thrombotic cardiac masses that resemble thrombi could not be excluded systematically.

Ascertainment of atrial fibrillation was based on routine clinical documentation and available electrocardiographic records. Systematic Holter monitoring or other prolonged ambulatory electrocardiographic monitoring was not performed for every patient. Therefore, paroxysmal atrial fibrillation may have been underdetected, and the recorded atrial fibrillation variable may not fully represent the true burden of atrial fibrillation. Because of this incomplete ascertainment, an atrial-fibrillation-adjusted sensitivity analysis was not considered sufficiently reliable and was not included. This limitation is relevant because atrial thrombus could not be excluded from the incompletely localized intracardiac-thrombus outcome; residual confounding by undetected atrial fibrillation and uncertainty regarding atrial versus ventricular thrombus location therefore cannot be ruled out.

Fourth, D-dimer was defined as the first measurement obtained at our hospital after AMI confirmation. The assay platform, reagent, reference interval, FEU/DDU convention, and possible platform changes during 2018–2025 were unavailable. Therefore, inter-year assay comparability and the validity of extreme individual values could not be independently verified. The D-dimer quartiles and cutoffs reported in this study should consequently be interpreted as cohort-specific descriptive categories rather than universal clinical thresholds. Linked timestamps between D-dimer sampling and the first thrombus-identifying examination were also unavailable, preventing a sensitivity analysis restricted to measurements obtained before thrombus detection. The association should therefore not be interpreted as prospective prediction or evidence that D-dimer preceded thrombus formation. Because admission year was used as the calendar-time variable, the calendar-time sensitivity analysis excluded patients admitted in 2025. Among patients with observed D-dimer measurements, the OR per D-dimer doubling was 1.25 in the full cohort and 1.50 after exclusion of patients admitted in 2025. The positive direction and statistical significance of the association were preserved, although the larger estimate and higher apparent AUC after exclusion indicate that calendar-time restriction influenced the magnitude of the observed association. This analysis was interpreted as a calendar-time sensitivity analysis and did not establish assay comparability across study years.

Fifth, aneurysm-location indicators were non-mutually exclusive and anterior and apical involvement frequently co-occurred. Although the patient-level cross-tabulation, phi coefficient, VIFs, apical-subgroup analysis, and complete-case analysis were performed, the anterior-involvement estimate should still be interpreted as a conditional association rather than an anatomically isolated or causal effect. Because D-dimer was measured during the index hospitalization and its temporal relationship to thrombus detection was not standardized, the extended laboratory-adjusted model should not be interpreted as establishing a causal or anatomically independent effect.

Finally, the apparent model-performance estimates were obtained in the development dataset without external validation, optimism correction, formal calibration assessment, or decision-curve analysis. The combined model showed only a minimal incremental apparent AUC of approximately 0.003 beyond the model containing D-dimer alone, and its clinical utility remains unestablished. The full-cohort and 2025-exclusion analyses used identical model specifications. However, because the sample sizes and observed D-dimer distributions differed, the resulting performance measures were interpreted descriptively rather than as evidence of a causal calendar-time effect.

## 5. Conclusions

Among hospitalized patients with AMI and ventricular aneurysm, higher D-dimer and anterior ventricular involvement were associated with echocardiographically documented intracardiac thrombus with incomplete chamber-specific localization. These findings concern contemporaneous associations and do not establish prospective prediction or causality. The anterior-involvement estimate should be interpreted as a conditional association in a cohort with extensive anterior–apical overlap, rather than as an anatomically isolated effect. Anterior involvement added only minimal apparent discrimination beyond the model containing D-dimer, and the clinical usefulness of the combined model remains unestablished.

## Figures and Tables

**Table 1:** Baseline characteristics of patients with ventricular aneurysm stratified by intracardiac thrombus status.

Characteristic	Overall (*n* = 1684)	No Intracardiac Thrombus (*n* = 1473)	Intracardiac Thrombus (*n* = 211)	*p* Value
Age, years	64.00 (55.00, 72.00)	64.00 (56.00, 72.00)	61.00 (52.00, 70.00)	<0.001
Male sex, *n* (%)	1271 (75.5%)	1090 (74.0%)	181 (85.8%)	<0.001
SBP, mmHg	129.00 (111.00, 148.00)	129.00 (111.00, 149.00)	128.00 (110.25, 142.00)	0.625
DBP, mmHg	82.00 (71.00, 95.00)	82.00 (71.00, 95.00)	82.00 (73.25, 95.75)	0.333
Pulse, beats/min	83.00 (73.00, 95.00)	83.00 (73.00, 95.00)	86.00 (76.00, 98.00)	0.002
History of hypertension, *n* (%)	913 (54.2%)	807 (54.8%)	106 (50.2%)	0.243
History of diabetes mellitus, *n* (%)	637 (37.8%)	551 (37.4%)	86 (40.8%)	0.388
History of atrial fibrillation, *n* (%)	134 (8.0%)	114 (7.7%)	20 (9.5%)	0.461
Prior stroke, *n* (%)	42 (2.5%)	35 (2.4%)	7 (3.3%)	0.559
NT-proBNP, pg/mL	2100.00 (947.57, 4758.75)	2044.00 (928.60, 4589.00)	2464.00 (1186.00, 5866.00)	0.016
hs-CRP, mg/L	6.00 (2.38, 10.00)	6.00 (2.25, 10.00)	6.10 (4.25, 10.00)	<0.001
Fibrinogen, g/L	3.96 (3.07, 5.08)	3.91 (3.04, 5.03)	4.22 (3.30, 5.42)	0.008
INR	1.06 (1.00, 1.14)	1.06 (1.00, 1.13)	1.08 (1.02, 1.17)	0.004
PTA, %	91.00 (80.00, 102.00)	91.30 (81.00, 102.22)	87.00 (76.00, 99.00)	<0.001
Albumin, g/L	37.10 (33.30, 40.88)	37.20 (33.50, 40.90)	36.50 (31.90, 40.50)	0.055
WBC, ×10^9^/L	8.84 (6.81, 11.44)	8.82 (6.82, 11.30)	8.95 (6.70, 12.51)	0.136
NEUT, ×10^9^/L	6.65 (4.83, 9.12)	6.65 (4.84, 9.02)	6.72 (4.81, 10.05)	0.266
LYM, ×10^9^/L	1.29 (0.93, 1.69)	1.29 (0.94, 1.69)	1.29 (0.89, 1.70)	0.920
Creatinine, μmol/L	72.00 (59.00, 92.00)	72.00 (59.00, 91.00)	77.00 (62.25, 96.75)	0.015
eGFR, mL/min/1.73 m^2^	90.41 (69.97, 102.19)	90.51 (70.33, 102.00)	90.11 (67.70, 103.49)	0.917
Glucose, mmol/L	6.17 (5.16, 8.40)	6.22 (5.19, 8.44)	5.74 (4.87, 8.08)	0.037
CK-MB, U/L	26.13 (15.00, 71.39)	27.00 (15.16, 77.38)	22.10 (14.68, 41.16)	0.002
cTnT, ng/mL	0.84 (0.16, 2.54)	0.86 (0.17, 2.54)	0.72 (0.15, 2.56)	0.627
Apical aneurysm, *n* (%)	1,610 (95.6%)	1,406 (95.5%)	204 (96.7%)	0.525
Anterior ventricular aneurysm, *n* (%)	784 (46.6%)	663 (45.0%)	121 (57.3%)	0.001
Inferior wall aneurysm, *n* (%)	51 (3.0%)	46 (3.1%)	5 (2.4%)	0.702
Septal aneurysm, *n* (%)	56 (3.3%)	49 (3.3%)	7 (3.3%)	1.000

Note: Values are presented as median (interquartile range) or *n* (%). *p* values were calculated using the Mann–Whitney U test for continuous variables and the chi-square test or Fisher’s exact test for categorical variables, as appropriate. Aneurysm locations were coded as linked patient-level, non-mutually exclusive indicators. D-dimer-specific results are provided in Supplementary Table S2. Abbreviations: SBP, systolic blood pressure; DBP, diastolic blood pressure; NT-proBNP, N-terminal pro-B-type natriuretic peptide; hs-CRP, high-sensitivity C-reactive protein; INR, international normalized ratio; PTA, prothrombin activity; WBC, white blood cell count; NEUT, neutrophil count; LYM, lymphocyte count; eGFR, estimated glomerular filtration rate; CK-MB, creatine kinase-MB; cTnT, cardiac troponin T.

**Table 2:** Performance of multivariable models for intracardiac thrombus among patients with ventricular aneurysm.

Model	N	Thrombus Events	AUC, Mean ± SD	ΔAUC vs. Base, Mean ± SD	DeLong *p* Range Across Imputations	Brier Score, Mean ± SD	AIC, Mean ± SD	BIC, Mean ± SD
Base clinical model	1684	211	0.635 ± 0.004	Reference	Reference	0.1066 ± 0.0002	1237.27 ± 2.40	1269.85 ± 2.40
Base + anterior ventricular aneurysm	1684	211	0.649 ± 0.003	0.014 ± 0.001	0.125–0.252	0.1057 ± 0.0001	1229.94 ± 2.07	1267.94 ± 2.07
Base + log D-dimer	1684	211	0.713 ± 0.003	0.078 ± 0.004	1.49 × 10^−9^–4.11 × 10^−8^	0.1049 ± 0.0002	1199.57 ± 2.58	1237.57 ± 2.58
Base + anterior ventricular aneurysm + log D-dimer	1684	211	0.716 ± 0.003	0.081 ± 0.004	5.14 × 10^−9^–1.01 × 10^−7^	0.1040 ± 0.0002	1190.64 ± 2.70	1234.08 ± 2.70

Note: Performance metrics were calculated separately in each of the 20 imputed datasets and are presented as arithmetic means ± standard deviations. DeLong tests were performed separately within each imputed dataset to compare each expanded model with the base clinical model. Because DeLong *p* values were not formally pooled across imputations, they are presented as descriptive ranges and should not be interpreted as pooled inferential estimates. AUC, area under the receiver operating characteristic curve; AIC, Akaike information criterion; BIC, Bayesian information criterion.

## Data Availability

The data underlying this article cannot be shared publicly due to the privacy of individuals who participated in the study. The data will be shared upon reasonable request to the corresponding author.

## Supplementary Materials

The following supporting information can be downloaded at https://www.mdpi.com/article/10.3390/jcm15208173/s1. Table S1: Segment-involvement associations with echocardiographically documented intracardiac thrombus compared with patients without ventricular aneurysm; Table S2: Association between D-dimer quartiles and echocardiographically documented intracardiac thrombus among patients with ventricular aneurysm; Table S3: Exploratory single-marker analyses of clinical indicators for echocardiographically documented intracardiac thrombus among patients with ventricular aneurysm; Table S4: Sequential multivariable models for anterior ventricular aneurysm and echocardiographically documented intracardiac thrombus; Table S5: Joint distribution of anterior and apical aneurysm involvement and echocardiographically documented intracardiac thrombus events; Table S6: Association and collinearity diagnostics for anterior and apical aneurysm indicators; Table S7: Sensitivity analyses restricted to patients with apical aneurysm; Table S8: Model complexity in the ventricular aneurysm cohort; Table S9: Calendar-year distribution of D-dimer measurements among patients with ventricular aneurysm; Table S10: D-dimer quartile boundaries and continuous-analysis specifications; Table S11: Sensitivity analysis of the association between continuous log2-transformed D-dimer and echocardiographically documented intracardiac thrombus under alternative handling of extreme recorded values; Table S12: Calendar-time sensitivity analysis of the association between continuous log2-transformed D-dimer and echocardiographically documented intracardiac thrombus; Table S13: Variable-specific missingness and imputation methods used in the multivariable analyses.
