# Supplementary material for: Association of Anterior Ventricular Aneurysm and D-Dimer with Intracardiac Thrombus in Patients with Acute Myocardial Infarction: A Retrospective Analysis
Source: J Clin Med. 2026 Oct 21;15(20):8173. doi: 10.3390/jcm15208173 (PMC13706533; doi:10.3390/jcm15208173)
Supplement: Supplementary file 1 [file jcm-15-08173-s001.zip › jcm-4510386-supplementary.pdf]

Association of Anterior Ventricular Aneurysm and D-Dimer with Intracardiac Thrombus in Patients with Acute Myocardial Infarction: A Retrospective Analysis

Bohan Li [1], Xiaorui Huang [1], Zizhu Lian [2], Shikang Liu [1], Tianyi Zhang [3], Tianjiao Lei [2], Guoliang Li [1], Yue Wu [1,*] and Chaofeng Sun [1,*]

[1] Department of Cardiovascular Medicine, The First Affiliated Hospital of Xi'an Jiaotong University, Xi'an 710061, China; xdtslbh@xjtu.edu.cn (B.L.); huangxr2025@163.com (X.H.); lsk@xjtufh.edu.cn (S.L.); liguoliang_med@163.com (G.L.)

[2] Department of Cardiovascular Surgery, The First Affiliated Hospital of Xi'an Jiaotong University, Xi'an 710061, China; lisalzz2651092799@163.com (Z.L.); ltj585@xjtufh.edu.cn (T.L.)

[3] Biobank, The First Affiliated Hospital of Xi'an Jiaotong University, Xi'an 710061, China; zhantianyi850214@xjtufh.edu.cn

[*] Correspondence: yue.wu@xjtu.edu.cn (Y.W.); cfsun1@xjtu.edu.cn (C.S.)

Supplementary Materials

Supplementary Table S1. Segment-involvement associations with echocardiographically documented intracardiac thrombus compared with patients without ventricular aneurysm

Aneurysm location	Patients, n	Documented intracardiac thrombus, n	Observed proportion, %	OR (95% CI) vs no ventricular aneurysm	P value
No ventricular aneurysm	16266	199	1.22	Reference	—
Anterior ventricular aneurysm	784	121	15.43	14.74 (11.60-18.71)	< 0.001
Apical aneurysm	1610	204	12.67	11.71 (9.56-14.35)	< 0.001
Septal aneurysm	56	7	12.50	11.53 (5.16-25.78)	< 0.001
Inferior wall aneurysm	51	5	9.80	8.78 (3.45-22.32)	< 0.001
Right ventricular aneurysm	2	0	0.00	Not estimated	—
Posterior wall aneurysm	1	0	0.00	Not estimated	—
Lateral wall aneurysm	1	0	0.00	Not estimated	—

Note: Patients without ventricular aneurysm were used as the reference group. Anatomical location variables were non-mutually exclusive because a patient could have aneurysmal involvement of more than one ventricular segment. These results are segment-involvement associations and should not be interpreted as direct comparisons or rankings between locations. Rare locations with no thrombus events were not estimated. OR, odds ratio; CI, confidence interval.

Supplementary Table S2. Association between D-dimer quartiles and echocardiographically documented intracardiac thrombus among patients with ventricular aneurysm

D-dimer quartile	N	Documented thrombus, n	Observed proportion, %	D-dimer, median (IQR), mg/L	Adjusted OR (95% CI)	P value
Q1	413	15	3.63	0.43 (0.34-0.51)	Reference	—
Q2	404	43	10.64	0.74 (0.68-0.82)	3.71 (2.01-6.83)	< 0.001
Q3	408	59	14.46	1.25 (1.06-1.49)	5.22 (2.88-9.44)	< 0.001
Q4	408	91	22.30	3.51 (2.37-7.15)	9.50 (5.30-17.00)	< 0.001

Note: Analyses included 1633 patients with an observed first D-dimer measurement obtained at our hospital after AMI confirmation; D-dimer values were not imputed. Quartile boundaries were 0.59, 0.91, and 1.80 mg/L and represent cohort-specific descriptive cut points, not validated clinical thresholds. Observed proportions denote the percentage of patients with echocardiographically documented intracardiac thrombus within each quartile. Adjusted ORs were estimated after adjustment for clinical variables, left ventricular systolic-function grade, other aneurysm locations, and laboratory markers; missing covariates were multiply imputed. These associations were exploratory. OR, odds ratio; CI, confidence interval.

Supplementary Table S3. Exploratory single-marker analyses of clinical indicators for echocardiographically documented intracardiac thrombus among patients with ventricular aneurysm

Indicator	OR per SD (95% CI)	P value	AUC (95% CI)	Best cutoff	Sensitivity, %	Specificity, %
D-dimer	1.04 (0.93-1.16)	0.465	0.677 (0.640-0.713)	0.78	82.0	46.2
Length of stay	1.31 (1.16-1.47)	< 0.001	0.633 (0.595-0.672)	3.70	77.3	44.5
apoA	0.72 (0.62-0.84)	< 0.001	0.596 (0.556-0.635)	1.15	86.3	28.6
PT	1.11 (0.99-1.24)	0.073	0.575 (0.535-0.615)	13.65	54.5	57.1
Age	0.76 (0.66-0.87)	< 0.001	0.575 (0.532-0.617)	56.50	38.9	73.3
HDL-C	0.79 (0.67-0.92)	0.003	0.572 (0.532-0.613)	0.89	48.4	64.2
C-reactive protein	1.27 (1.09-1.47)	0.002	0.571 (0.531-0.610)	5.99	65.0	47.2
PTR	1.13 (1.01-1.26)	0.035	0.567 (0.526-0.609)	1.05	59.3	50.8
CK-MB	0.78 (0.64-0.95)	0.015	0.566 (0.527-0.605)	34.01	73.0	40.9
Uric acid	1.27 (1.11-1.46)	< 0.001	0.565 (0.522-0.608)	361.50	46.0	68.0
Blood urea nitrogen	1.20 (1.07-1.35)	0.002	0.564 (0.522-0.606)	5.77	53.6	63.7
International normalized ratio	1.12 (1.00-1.25)	0.045	0.563 (0.521-0.604)	1.06	59.2	50.9
Fibrinogen	1.21 (1.05-1.39)	0.007	0.559 (0.518-0.600)	3.88	61.1	50.6
Indirect bilirubin	0.84 (0.71-1.00)	0.046	0.550 (0.508-0.592)	7.95	41.2	68.4
Cholinesterase	0.85 (0.74-0.99)	0.032	0.547 (0.505-0.590)	6328.00	47.4	61.9
Albumin	0.84 (0.72-0.97)	0.017	0.540 (0.497-0.584)	32.45	28.4	81.5
Neutrophil count	1.19 (1.04-1.35)	0.012	0.524 (0.480-0.568)	9.89	28.4	80.7

Note: Candidate-marker analyses were exploratory and included 78 markers evaluated using single-marker AUC and univariable analyses. These analyses were not treated as validation of a clinical prediction model. Because data-driven marker evaluation can introduce optimism, the final adjusted models and apparent performance comparisons should be interpreted cautiously. AUC, area under the curve; OR, odds ratio; CI, confidence interval.

Supplementary Table S4. Sequential multivariable models for anterior ventricular aneurysm and echocardiographically documented intracardiac thrombus

Model	Adjustment	OR (95% CI)	P value	N	Events
Model 1: unadjusted	Unadjusted	1.64 (1.23-2.20)	< 0.001	1684	211
Model 2: adjusted for other aneurysm locations	Adjusted for other aneurysm locations	1.67 (1.23-2.25)	< 0.001	1684	211
Model 3: adjusted for clinical variables	Adjusted for clinical variables and other aneurysm locations	1.63 (1.20-2.21)	0.002	1684	211
Model 4: extended exploratory model including laboratory markers	Extended exploratory model including clinical variables, aneurysm locations, and laboratory markers	1.65 (1.20-2.26)	0.002	1684	211

Note: Sequential logistic regression models were constructed within the ventricular aneurysm cohort and compared patients with versus without anterior ventricular involvement. Model 1 was unadjusted. Model 2 adjusted for apical, inferior, and septal aneurysm involvement. Model 3 additionally adjusted for age, sex, systolic blood pressure, pulse, and ordinal left ventricular systolic-function grade. Model 4 was prespecified as an extended exploratory model and additionally included log2-transformed D-dimer, log2-transformed NT-proBNP, log2(hs-CRP + 1), albumin, fibrinogen, INR, prothrombin activity, and eGFR. Because the temporal relationship between D-dimer measurement and thrombus detection was not standardized, Model 4 was not interpreted as a causal fully adjusted model. Lateral, posterior, and right ventricular aneurysm indicators were excluded because they were extremely uncommon and had no thrombus events. Missing model covariates were handled in 20 multiply imputed datasets; Model 4 was fitted in each dataset, and regression coefficients, standard errors, 95% CIs, and P values were pooled using Rubin's rules. Predictor-parameter counts and events-per-parameter ratios are reported in Supplementary Table S8.

Supplementary Table S5. Joint distribution of anterior and apical aneurysm involvement and echocardiographically documented intracardiac thrombus events

Anterior-apical combination	Patients, n	Thrombus events, n	Event proportion, %
Neither anterior nor apical	64	7	10.9
Apical only	836	83	9.9
Anterior only	10	0	0.0
Both anterior and apical	774	121	15.6

Note: Counts are patient-level combinations among 1684 patients with ventricular aneurysm. Event proportions were calculated within each anterior-apical combination. Location indicators were non-mutually exclusive.

Supplementary Table S6. Association and collinearity diagnostics for anterior and apical aneurysm indicators

A. Anterior-apical association

Metric	Value	Interpretation
Phi correlation coefficient	0.142	Weak positive correlation
Chi-square P value	< 0.001	Anterior and apical indicators were associated
P (apical ∣ anterior)	98.7%	Proportion of anterior cases with apical involvement
P (anterior ∣ apical)	48.1%	Proportion of apical cases with anterior involvement

B. Variance inflation factors across 20 multiply imputed datasets

Variable	Median VIF	VIF range	Maximum VIF
Anterior involvement	1.06	1.06-1.06	1.06
Age	1.34	1.33-1.35	1.35
Sex	1.13	1.13-1.14	1.14
Systolic blood pressure	1.02	1.01-1.03	1.03
Pulse	1.04	1.04-1.04	1.04
LV systolic-function grade	1.05	1.05-1.06	1.06
Apical involvement	2.19	2.18-2.19	2.19
Inferior involvement	1.98	1.98-1.99	1.99
Septal involvement	1.30	1.29-1.30	1.30
Log D-dimer	1.14	1.13-1.15	1.15
log2-transformed NT-proBNP	1.81	1.79-1.85	1.85
Log hs-CRP	1.39	1.38-1.41	1.41
Albumin	1.26	1.25-1.28	1.28
Fibrinogen	1.36	1.35-1.38	1.38
INR	2.06	2.04-2.08	2.08
Prothrombin activity	2.24	2.21-2.28	2.28
eGFR	1.50	1.45-1.51	1.51

Note: VIFs were calculated separately using the final-model design matrix in each of 20 imputed datasets and are summarized as the median, range, and maximum. The largest observed VIF was 2.28. VIF, variance inflation factor; NT-proBNP, B-type natriuretic peptide; hs-CRP, C-reactive protein; INR, international normalized ratio; eGFR, estimated glomerular filtration rate.

Supplementary Table S7. Sensitivity analyses restricted to patients with apical aneurysm

Analysis	N	Events	OR (95% CI)	P value	Adjustment
Crude	1610	204	1.68 (1.25-2.27)	< 0.001	None
Extended exploratory	1610	204	1.71 (1.24-2.36)	< 0.001	Extended exploratory model covariates except apical involvement
Complete-case adjusted	508	73	1.86 (1.08-3.25)	0.026	Same covariates; complete cases

Note: All analyses compared patients with versus without additional anterior involvement within the apical-aneurysm subgroup. The extended exploratory model included age, sex, systolic blood pressure, pulse, ordinal left ventricular systolic-function grade, inferior and septal aneurysm involvement, log2-transformed D-dimer, log2-transformed NT-proBNP, log2(hs-CRP + 1), albumin, fibrinogen, INR, prothrombin activity, and eGFR. The apical indicator was omitted because it was invariant; rare lateral, posterior, and right ventricular indicators were not included. OR, odds ratio; CI, confidence interval.

Supplementary Table S8. Model complexity in the ventricular aneurysm cohort

Model	Predictor parameters	Events	Events per parameter	Specification
Model 1	1	211	211.0	Anterior involvement
Model 2	4	211	52.8	Model 1 plus apical, inferior, and septal involvement
Model 3	9	211	23.4	Model 2 plus five clinical covariates
Model 4	17	211	12.4	Model 3 plus eight laboratory covariates

Note: Parameter counts exclude the intercept and assume the reported one-parameter coding for each binary, continuous, log-transformed continuous, and ordinal covariate. Events per predictor parameter were calculated as 211 divided by the corresponding parameter count. These ratios are descriptive and do not by themselves demonstrate sample-size adequacy or exclude overfitting.

Supplementary Table S9. Calendar-year distribution of D-dimer measurements among patients with ventricular aneurysm

Year	Cohort n	Measured n	Events	Median (IQR), mg/L	P95	Maximum	>10 mg/L	>100 mg/L	>1000 mg/L
2018	87	85	13	1.00 (0.70-1.90)	8.81	26.83	3	0	0
2019	121	119	22	0.82 (0.50-1.60)	6.15	27.70	3	0	0
2020	91	83	11	0.78 (0.47-1.56)	6.54	11.80	4	0	0
2021	160	156	27	0.80 (0.39-1.81)	11.97	85.72	10	0	0
2022	251	245	24	0.82 (0.50-1.93)	7.89	29.29	9	0	0
2023	341	338	52	0.97 (0.71-1.85)	5.11	46.82	4	0	0
2024	369	360	32	0.93 (0.59-1.66)	7.32	28.60	12	0	0
2025	264	247	30	1.03 (0.66-2.46)	410.47	11021.90	27	20	7

Note: D-dimer values were recorded in the analytic database in mg/L. This table presents the observed distribution by admission year and does not verify the assay platform, reference interval, FEU/DDU reporting convention, or changes in laboratory methods during the study period. The calendar-year distributions are descriptive and should not be interpreted as evidence of assay comparability across years. The event counts in this table refer to echocardiographically documented thrombus events in the full year-specific ventricular-aneurysm cohort and are not restricted to patients with observed D-dimer measurements.

Supplementary Table S10. D-dimer quartile boundaries and continuous-analysis specifications

Component	Specification
Observed quartile boundaries	Q1/Q2 0.59 mg/L, Q2/Q3 0.91 mg/L, Q3/Q4 1.80 mg/L
Continuous transformation	log2(D-dimer); OR interpreted per doubling
Per-doubling association	OR 1.24 (95% CI 1.15-1.35), P<0.001
Adjusted imputed continuous model	OR 1.24 (95% CI 1.14-1.34), P<0.001
Complete-case model	OR 1.33 (95% CI 1.09-1.62), P=0.005
Spline internal knots	0.69 and 1.40 mg/L
Spline boundary knots	0.05 and 11021.90 mg/L
Spline reference	0.91 mg/L

Note: Quartile boundaries were recalculated from observed patient-level values. Spline estimates and confidence intervals are exploratory and should not be interpreted as validated clinical thresholds.

Supplementary Table S11. Sensitivity analysis of the association between continuous log2-transformed D-dimer and echocardiographically documented intracardiac thrombus under alternative handling of extreme recorded values

**Extreme-value handling scenario**	**N**	**Thrombus events**	**Excluded observations**	**OR per D-dimer doubling (95% CI)**	**P value**	**Mean AUC**	**Mean Brier score**
Original recorded values	1633	208	0	1.24 (1.14-1.34)	< 0.001	0.734	0.1032
Exclusion of the upper 1%	1616	206	17	1.48 (1.32-1.67)	< 0.001	0.749	0.1018
Winsorization at the 99th percentile	1633	208	0	1.29 (1.18-1.41)	< 0.001	0.736	0.1030
Exclusion of recorded values >100 mg/L	1613	205	20	1.50 (1.32-1.70)	< 0.001	0.750	0.1016
Exclusion of recorded values >1000 mg/L	1626	206	7	1.30 (1.18-1.43)	< 0.001	0.737	0.1024

Note: Analyses were conducted among 1633 patients with observed D-dimer measurements and 208 thrombus events. Odds ratios are reported per doubling of D-dimer with 95% confidence intervals. The same model covariates and missing-data procedure as in the primary analysis were applied where applicable. Extreme-value handling consisted of excluding the upper 1%, winsorizing values at the 99th percentile, excluding recorded values >100 mg/L, or excluding recorded values >1000 mg/L. These thresholds were exploratory stress-test definitions and were not interpreted as assay-specific clinical cutoffs. AUC and Brier score are apparent performance measures calculated in the study dataset. OR, odds ratio; CI, confidence interval; AUC, area under the receiver operating characteristic curve.

Supplementary Table S12. Calendar-time sensitivity analysis of the association between continuous log2-transformed D-dimer and echocardiographically documented intracardiac thrombus.

Scenario	N with observed D-dimer	Thrombus events	OR per D-dimer doubling (95% CI)	P value	AUC, mean ± SD	Brier score, mean ± SD	AIC, mean ± SD	BIC, mean ± SD
Full cohort	1633	208	1.25 (1.15–1.35)	<0.001	0.726 ± 0.003	0.1034 ± 0.0002	1166.26 ± 3.18	1263.43 ± 3.18
Excluding patients admitted in 2025	1386	179	1.50 (1.31–1.72)	<0.001	0.749 ± 0.001	0.1021 ± 0.0002	975.55 ± 1.37	1069.77 ± 1.37

Note: Both rows were estimated using the same prespecified adjusted continuous log2-transformed D-dimer model, including the prespecified clinical, aneurysm-location, and laboratory covariates. The only difference was the exclusion of patients admitted in 2025 according to the hospital admission date. This sensitivity model was distinct from the simpler Base + log D-dimer model used in Table 2. ORs represent the change in odds per doubling of D-dimer. AUC, Brier score, AIC, and BIC are presented as arithmetic means ± standard deviations across 20 imputed datasets and were interpreted as exploratory apparent-performance measures. Because the cohort sizes and observed D-dimer distributions differed, AIC and BIC were reported descriptively and were not used for direct between-cohort comparison. The analysis was not intended to establish assay comparability across calendar years. OR, odds ratio; CI, confidence interval; AUC, area under the receiver operating characteristic curve; AIC, Akaike information criterion; BIC, Bayesian information criterion.

Supplementary Table S13. Variable-specific missingness and imputation methods used in the multivariable analyses

Variable	Role / scale	Missing, n (%)	Imputation method	Included in predictor matrix	Transformation before imputation
Echo-documented intracardiac thrombus (outcome)	Binary outcome	0 (0.0)	Not imputed	Not applicable; outcome was included as a predictor for covariate imputation	None
Anterior aneurysm	Binary	0 (0.0)	Logistic regression (logreg)	Yes	None
Apical aneurysm	Binary	0 (0.0)	Logistic regression (logreg)	Yes	None
Inferior aneurysm	Binary	0 (0.0)	Logistic regression (logreg)	Yes	None
Septal aneurysm	Binary	0 (0.0)	Logistic regression (logreg)	Yes	None
Male sex	Binary	0 (0.0)	Logistic regression (logreg)	Yes	None
Age	Continuous	0 (0.0)	Predictive mean matching (PMM)	Yes	None
Systolic blood pressure	Continuous	81(4.81)	Predictive mean matching (PMM)	Yes	None
Pulse rate	Continuous	12 (0.71)	Predictive mean matching (PMM)	Yes	None
Left ventricular systolic-function grade	Ordinal covariate treated numerically in the model	37 (2.20)	Predictive mean matching (PMM)	Yes	None
log2-transformed D-dimer	Continuous	51 (3.03)	Predictive mean matching (PMM)	Yes	log2(D-dimer), before imputation
log2-transformed NT-proBNP	Continuous	39 (2.32)	Predictive mean matching (PMM)	Yes	log2(BNP), before imputation
log2-transformed hsCRP	Continuous	147 (8.73)	Predictive mean matching (PMM)	Yes	log2(hs-CRP + 1), before imputation
Albumin	Continuous	58 (3.44)	Predictive mean matching (PMM)	Yes	None
Fibrinogen	Continuous	39 (2.32)	Predictive mean matching (PMM)	Yes	None
INR	Continuous	38 (2.26)	Predictive mean matching (PMM)	Yes	None
Prothrombin activity	Continuous	38 (2.26)	Predictive mean matching (PMM)	Yes	None
eGFR	Continuous	168 (9.98)	Predictive mean matching (PMM)	Yes	None

Note: The analytic cohort comprised 1,684 patients. Twenty imputed datasets were generated using multiple imputation by chained equations, with 20 iterations per dataset. Binary variables were imputed using logistic regression and continuous variables using predictive mean matching. The outcome indicator and all prespecified model variables were included in the predictor matrix. Log2-transformed D-dimer, log2-transformed NT-proBNP, and log2(hs-CRP + 1) were generated before imputation and imputed on the transformed scale. A ridge parameter of 1 × 10−4 was used for numerical stability. Variables without missing values were not actually imputed, although their prespecified methods are shown for completeness. Trace and density plots were visually inspected; no obvious systematic drift or substantial incompatibility between observed and imputed distributions was identified. Missingness percentages were calculated using the full analytic cohort.

Abbreviations: NT-proBNP, N-terminal pro-B-type natriuretic peptide; hs-CRP, high-sensitivity C-reactive protein; eGFR, estimated glomerular filtration rate; INR, international normalized ratio; PMM, predictive mean matching.
